# Strengthening Student Nurses’ Clinical Learning in Greece Through Mentorship: Findings from a Narrative Review and a National Stakeholder Focus Group

**DOI:** 10.3390/nursrep15120445

**Published:** 2025-12-11

**Authors:** Stefanie Praxmarer-Fernandes, Eleni Roditi, Theodoros Katsoulas, Brigita Skela-Savič, Margrieta Langins, Christos Triantafyllou, Joao Breda

**Affiliations:** 1 WHO Regional Office for Europe, 2100 Copenhagen, Denmark; 2 Department of Nursing, National and Kapodistrian University of Athens, 11527 Athens, Greece; 3 Angela Boškin Faculty of Health Care, 4270 Jesenice, Slovenia; 4 WHO Athens Quality of Care and Patient Safety Office, 10675 Athens, Greece

**Keywords:** Greece, nursing education, mentorship, clinical instruction, clinical education, workforce development, focus group

## Abstract

**Background/Objectives:** Clinical instruction and mentorship are essential components of nursing education and early professional development. In Greece, while nursing curricula align with EU directives mandating both theoretical and clinical training, significant gaps persist in the quality, coordination, and legislative support of mentorship. This work aims to (i) synthesise evidence on clinical instruction and mentorship in Greece and draw on selected European examples to provide contextual insight, and (ii) integrate national stakeholder perspectives to generate actionable recommendations for a Greek clinical mentorship framework. **Methods**: A narrative literature review was conducted, identifying 19 eligible articles examining mentorship, clinical instruction and preceptorship in European and Greek contexts. In addition, a national stakeholder focus group with 25 participants, including representatives from academia, healthcare institutions, regulatory bodies, and nursing associations, was held in Athens in 2024. Data from both sources were thematically analysed and integrated to identify gaps, best practices, and context-specific recommendations. **Results**: Findings revealed inconsistent collaboration between universities and clinical institutions, limited training and recognition for clinical instructors, and the absence of a unified national framework. Stakeholders highlighted structural barriers to clinical mentoring such as understaffing and lack of policy support and expressed strong interest in a mentorship reform. Comparative analysis with European models demonstrated feasible pathways for Greece, including structured training, certification, and non-financial incentives. During the national stakeholder focus group, a dual-pathway mentorship system tailored for nursing students and newly hired nurses was most recommended to ensure both continuity and quality in professional development of nurses. **Conclusions**: Despite alignment with EU directives, Greece lacks an integrated national mentorship framework that ensures consistent clinical learning and supports workforce development. Two priority policy actions emerge from this work: (1) establishing a legally supported national certification and training system for clinical mentorship, and (2) educational structures in the clinical setting to improve educational quality, workforce retention and patient care outcomes.

## 1. Introduction

Clinical mentorship is a key element of nursing education and practice, where clinical instructors support the integration of theoretical knowledge into safe and competent clinical care. Clinical instructors, often functioning as mentors, guide student nurses and newly recruited nurses in developing confidence, critical thinking, and professional identity [1,2,3]. Preceptorship offers complementary, structured support during transition periods, particularly from student to registered nurse [4,5,6].

Across Europe, reforms have emphasised harmonised nursing education, bachelor-level preparation, and structured clinical mentorship models, aligned with the Bologna Process and EU workforce strategies [7,8,9]. Studies consistently show that higher proportions of bachelor-prepared nurses are associated with improved patient outcomes, including reductions in mortality and adverse events; for example, every 10% increase in bachelor-educated nurses corresponds to a 7% reduction in 30-day inpatient mortality [10,11,12]. These findings illustrate the broader importance of well-prepared graduates and reinforce the need for high-quality clinical learning environments, including structured mentorship. Global frameworks reinforce the importance of mentorship. The WHO Global Strategic Directions for Nursing and Midwifery identify clinical mentorship as essential for workforce capacity and patient safety [13], while Benner’s Novice-to-Expert model underscores the developmental nature of clinical competence [14], and Duchscher’s transition shock describes the vulnerability of early-career nurses [15].

In Greece, Registered Nurses complete four-year bachelor programmes aligned with EU Directive 2005/36/EC [16] including 2300 h each of theoretical and clinical training. Although collaboration between universities and healthcare institutions is mandated, its implementation varies widely [17,18]. Clinical instructors often lack formal preparation, recognition, and institutional support, and existing legislation remains fragmented [19,20]. A 2023 decree outlining requirements for clinical instructors represents progress, but systematic implementation has yet to occur.

Despite alignment with EU standards, Greece currently operates without a coordinated system to guide clinical mentorship. This gap limits quality assurance in clinical education and contributes to variability in student and new-graduate experiences.

Even though there is a growing body of international evidence, there remains little consolidated knowledge on how clinical mentorship is currently structured and experienced within Greece, nor how Greek practices compare with established European models. Existing Greek studies primarily examine isolated aspects of clinical training, but no prior work has integrated national stakeholder insights with a narrative synthesis to identify system-level gaps and feasible pathways for reform. This project addresses this knowledge gap by analysing both the literature and stakeholder perspectives to inform a coherent, context-appropriate approach to clinical mentorship in Greece.

Therefore, the aim of this work is to synthesise evidence on clinical mentorship in Greece and draw on selected European examples to contextualise potential approaches, and to integrate national stakeholder perspectives to inform the development of a coherent, context-appropriate toolkit for clinical mentorship. To achieve this aim, the project addresses the following research questions:

RQ1: What are the current strengths and gaps in clinical mentorship in Greece?

RQ2: What policy and organisational actions do stakeholders prioritise for strengthening clinical mentorship in Greece?

This is the first analysis to combine a narrative synthesis of the literature with a multi-stakeholder national focus group to propose a dual-pathway mentorship model tailored to nursing students and newly hired nurses. The primary unit of analysis is clinical mentorship for undergraduate nursing students and early-career nurses in Greek hospitals. The intended audience includes policy makers, university leaders, healthcare organisations, and nurse managers responsible for education, workforce development, and practice governance.

This project synthesises evidence from a narrative review of Greek and European literature alongside insights from a national multi-stakeholder focus group to examine clinical mentorship for undergraduate students and early-career nurses in Greece. The literature focuses primarily on student mentorship, while the stakeholder consultation expands the analysis to challenges affecting newly hired nurses. Together, these sources inform context-specific recommendations and a dual-pathway approach to strengthening clinical mentorship. The project addresses two research questions: (1) What are the current strengths and gaps in clinical mentorship in Greece? and (2) What policy and organisational actions do stakeholders prioritise to support improvement? Therefore, while the evidence base is centred on student mentorship, the stakeholder perspectives broaden the analysis to include issues relevant for both nursing students and early-career nurses within the Greek context.

## 2. Materials and Methods

### 2.1. Approach and Components of the Project

This project formed part of a WHO-supported technical activity under the Health-IQ initiative and integrated two complementary sources of evidence: (i) a narrative review of literature, and (ii) a structured national stakeholder consultation. This work was conducted within the broader strategic context of the WHO European Region priorities for 2021–2025, but these priorities served only as background and did not determine the analytical framework used. The aim was not to conduct a systematic comparative analysis of European mentorship frameworks. Instead, the narrative review selectively incorporated illustrative international examples (e.g., England, Finland, The Netherlands) to provide contextual benchmarks relevant for Greece. The consultation aimed to gather expert perspectives rather than collect personal or sensitive data. As this was a professional stakeholder engagement activity and not human-subjects research, formal institutional review board approval was not required. Notes taken during the consultation were anonymised and summarised to identify key themes relevant to mentoring structures, system gaps, and opportunities for improvement. The qualitative approach was selected to capture the complexity of clinical mentorship practices, gaps, and opportunities in the Greek nursing education system, rather than to test hypotheses. The descriptive orientation allowed for synthesis of both published evidence and experiential perspectives, resulting in a broad yet context-sensitive understanding of the issue.

Data integration was guided by principles of Qualitative Comparative Analysis (QCA). Although not used here in its formal Boolean application, QCA informed the analytic framework by encouraging examination of how different combinations of factors, such as institutional support, training availability, and legislative context, shape outcomes in clinical mentorship. This made it possible to identify context-specific influences on mentorship effectiveness and highlight potential pathways for reform tailored to Greece’s healthcare and educational realities.

### 2.2. Narrative Review

A narrative literature review was conducted to map existing knowledge and identify gaps concerning clinical mentorship in nursing education. This evidence was used to contextualise the Greek findings; however, due to variability in available detail across countries, a structured cross-country comparison was not feasible. Instead, key transferable elements from international models were synthesised narratively. Narrative synthesis was chosen because it allows integration of findings from diverse sources, including empirical research, conceptual papers, and grey literature, while accommodating differences in evidence and terminology.

Searches were carried out in PubMed and Google Scholar in both English and Greek. Keywords included “mentorship,” “preceptorship,” “clinical instructor,” “nursing education,” “Europe,” and “Greece.” The search covered publications from January 2010 to December 2024, ensuring inclusion of both recent reforms and longer-term trends in nursing education.

Grey literature sources included national policy documents, regulatory guidelines, ministerial reports, professional association publications, WHO reports, and institutional frameworks related to clinical training in Greece and Europe. These sources were retrieved from government websites, nursing regulatory bodies, and international organisations.

**Figure 1:**
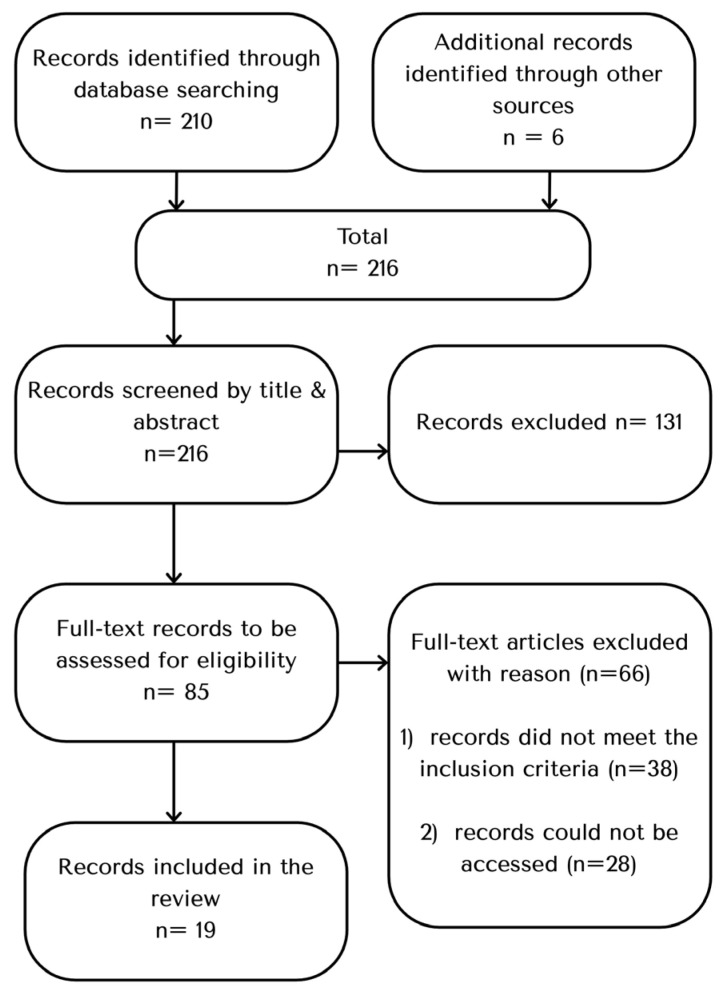
Flow diagram of the narrative review search and screening process.

In total, 216 records were retrieved (210 through database searching and 6 through other sources). After screening of titles and abstracts, 131 records were excluded. Eighty-five full-text articles were assessed for eligibility, of which 66 were excluded (38 did not meet the inclusion criteria; 28 could not be accessed). Nineteen full-text records were included in the narrative review. Figure 1 shows narrative review search and screening process. Appendix A shows the 19 full-text records. Two reviewers (T.K. and E.R.) independently conducted the search, screened titles and abstracts, and assessed full texts for eligibility. Any discrepancies were resolved through discussion and consensus with a third reviewer (S.PF).

Inclusion criteria comprised publications examining clinical instruction, mentorship, or preceptorship in undergraduate or early-career nursing education within European or Greek contexts. Articles without relevance to “education” or “mentorship” were defined as publications that did not address clinical instruction, mentorship, preceptorship, or supervisory roles in undergraduate or early-career nursing. Studies focusing on unrelated clinical topics (e.g., disease management, patient outcomes without educational components), other health professions, or general workforce issues without a link to training or mentorship were excluded. Relevance was determined independently by two reviewers based on title, abstract, and, when unclear, full-text assessment. Data were extracted and synthesised narratively under the following thematic categories:

(1) definitions and terminology,
(2) European-level reforms influencing clinical education and mentorship,
(3) mentorship roles, responsibilities, and transition-to-practice support,
(4) preparation, training, and recognition of clinical instructors, and
(5) policy and organisational gaps affecting mentorship implementation.

### 2.3. Stakeholder Focus Group

To complement literature findings with context-specific insights, a national focus group meeting was convened in Athens in November 2024. Thirty-one invitations were issued through purposive sampling to stakeholders from universities, tertiary hospitals, primary healthcare centres, professional nursing associations, student and early-career nurse organisations, regulators, and policy authorities. Twenty-five stakeholders attended.

The focus group was structured as a half-day workshop. Stakeholders were divided into four moderated groups of 5–8 members each. Discussions followed a semi-structured framework covering eight domains: (1) current mentorship roles and responsibilities, (2) support systems and enablers, (3) training and professional development, (4) collaboration between academic and clinical settings, (5) recognition and incentives, (6) institutional and policy barriers, (7) cultural factors, and (8) sustainability and innovation. Moderators facilitated balanced dialogue, while trained note-takers captured detailed records. Discussions were held in Greek, with real-time English interpretation available for summary purposes.

### 2.4. Data Analysis

Data were collected through structured notes, which were consolidated immediately after the workshop. These were analysed using open coding, with responses grouped into overarching categories and themes. Two researchers independently coded the data. Given the small dataset, formal inter-coder reliability statistics were not calculated. Instead, a systematic reconciliation process was applied: both coders compared their codes line-byline, discussed discrepancies, and agreed on a final coding structure through consensus. This approach ensured interpretive alignment and strengthened the dependability of the findings. To enhance reliability, the analytic process followed a structured QCA-informed approach. Coding decisions were cross-checked by two members of the project team, with discrepancies resolved through consensus.

Integration of evidence from the literature review and the stakeholder meeting served as an additional form of triangulation to strengthen the consistency of interpretations. All coding was conducted manually using open coding techniques. No qualitative software was used, as the dataset consisted of structured notes summarised immediately after the stakeholder discussion. A preliminary codebook was developed deductively from the discussion guide and inductively from repeated reading of the notes. Codes were grouped into overarching categories, which formed the basis of the thematic structure. Appendix B provides examples of theme identification. The stakeholder discussion was conducted primarily in Greek, with simultaneous English interpretation provided only for non-Greek-speaking attendees during whole-group exchanges. During small-group work, each group included a bilingual facilitator who supported communication and ensured accuracy. Notes were recorded in both Greek and English and subsequently reviewed by a second bilingual team member to verify meaning and minimise interpretive loss.

### 2.5. Ethical Considerations

The focus group was convened with the explicit aim of informing the development of a national mentorship toolkit for Greece. Stakeholders were invited on this basis and engaged with full awareness of this purpose. Because this activity was conducted as part of a national policy-development process rather than human-subjects research, institutional ethics review was not required. However, all participants provided written informed consent to participate and to allow the use of their anonymised contributions in the publication. To safeguard confidentiality, only aggregated data are reported.

### 2.6. Data Integration

Findings from the narrative literature review and the focus group were brought together through an iterative process of comparison and synthesis. Thematic categories derived from the literature, such as definitions, mentorship roles, training, incentives, and policy frameworks, were used as an initial framework to guide integration. Focus group notes were then coded against these categories, while allowing new themes to emerge inductively.

To situate the Greek findings within a broader context, the synthesised results were compared with mentorship models reported in selected European countries (e.g., United Kingdom, Finland, The Netherlands). This comparative step helped identify transferable practices and highlight areas where Greece faces unique structural challenges.

The integration process was guided by principles of Qualitative Comparative Analysis (QCA), not in a formal Boolean sense, but as a conceptual tool to explore how combinations of factors, such as institutional support, legislation, and training availability, influence the effectiveness of clinical mentorship. Researcher triangulation and peer debriefing within the project team were used to enhance the credibility and trustworthiness of the synthesis.

This project did not involve human or animal intervention and therefore did not require formal ethical approval.

## 3. Results

### 3.1. Literature Review Findings

In this review, findings from 19 records were included, with the full list provided in Appendix A. The results are organised into four themes:

Theme 1: System-level factors in European nursing education and mentorship.
Theme 2: Definitions and conceptual variations in mentorship models.
Theme 3: Transition to practice and early-career vulnerability.
Theme 4: Preparation and attributes of effective clinical instructors.

Appendix C provides a comparative overview of the clinical mentorship and education frameworks in the European countries discussed in the narrative review below, summarising their regulatory structures, mentor preparation requirements, and supervision models.

Theme 1: System-level factors in European nursing education and mentorship

Across European countries, nursing reforms have prioritised the harmonisation of education systems, bachelor-level qualifications as the entry standard, and the implementation of structured mentorship models [7,8,9]. These reforms are associated with broader strategies on professional mobility, patient safety, and workforce quality. Evidence indicates that higher proportions of bachelor-prepared nurses are linked with improved patient outcomes; for example, Aiken et al. (2011) reported a 7% reduction in 30-day inpatient mortality for every 10% increase in bachelor-educated nurses [21]. Additional studies show reductions in medication errors, falls, failure-to-rescue events, and improved patient experiences [10,12,21].

Theme 2: Definitions and conceptual variations in mentorship models

The literature demonstrates inconsistent use of terms such as “mentorship,” “preceptorship,” and “clinical instruction,” with varying emphases across studies. Mentorship is commonly characterised as a long-term developmental relationship that includes emotional support, guidance, and professional identity formation [3,22]. Preceptorship tends to be short-term and task-focused, frequently attached to newly qualified nurses or transition periods [4,5]. Clinical instruction often operates as an umbrella term integrating elements of both approaches in undergraduate programmes [23,24]. This inconsistency in terminology complicates comparison across educational systems. A summary of key distinctions between these concepts is provided in Table 1 to assist reader’s clarity.

Theme 3: Transition to practice and early-career vulnerability

The first year of practice is described as a period of heightened vulnerability for new graduates, commonly referred to as “transition shock” [15]. Without structured support, this phase is marked by stress, low confidence, and increased attrition. The novice-to-expert model outlines how nurses develop competence through experience [14], and studies report that mentorship and transition programmes can reduce turnover and burnout while strengthening professional commitment and resilience [25,26,27,28].

Theme 4: Preparation and attributes of effective clinical instructors

The literature highlights the importance of structured preparation for clinical instructors. Core attributes include communication skills, leadership, adult learning competence, and the ability to model professional behaviour [24,29]. European countries have introduced national certification programmes, training requirements, and incentive structures to support instructor readiness. Reported incentives include promotion credits, continuing education opportunities, protected time, reduced workload, and financial rewards [9,23]. Evidence from England, Finland, and The Netherlands demonstrates that structured approaches improve student learning outcomes, increase mentor satisfaction, and strengthen retention [9,23].

A summary of the key findings from each theme is presented in Table 2.

These patterns identified in the literature informed the design of the focus group, which further examined how clinical mentorship challenges and opportunities are experienced by stakeholders in Greece.

### 3.2. Focus Group Findings

Theme 5: Terminology and role clarity

Consultation findings highlighted inconsistent use of terminology, with overlapping references to “mentor,” “clinical instructor,” and “clinical coordinator.” Responsibilities typically included supervision, evaluation, and pastoral support, but these were often informal and dependent on individual initiative rather than structured assignment. Clinical mentor assignment processes were described by most stakeholders as informal and contingent on daily staff availability, leading to a lack of consistency.

Theme 6: Support and training needs

Synthesised stakeholder input showed a strong agreement on the need for a national training curriculum for clinical instructors. Suggested content included adult learning theory, leadership, communication, simulation-based teaching, and evaluation methods. Clinical mentors reported feeling inadequately prepared without structured training. There was agreement in all groups that structured preparation is essential, as mentors require appropriate tools and training to guide students effectively.

Theme 7: Academic–clinical collaboration

While formal agreements between universities and hospitals exist, implementation was described as inconsistent and largely symbolic. Stakeholders stressed the need for joint curricula, common selection criteria for instructors, and shared accountability mechanisms. The discussions highlighted ongoing fragmentation between academic institutions and clinical settings, with most groups noting that formal agreements often do not translate into practical collaboration.

Theme 8: Incentives and recognition

Aggregated stakeholder reflections highlighted that direct financial incentives are not considered feasible under existing health system constraints. Instead, non-financial motivators such as professional recognition, continuing education credits, reduced night shifts, and the integration of mentorship into career advancement frameworks were consistently identified as more feasible and sustainable approaches to supporting mentorship roles. Several participants highlighted that meaningful recognition and clear opportunities for career advancement are viewed as stronger motivators than small financial payments.

Theme 9: Structural and organisational barriers

Chronic understaffing, time constraints, cultural resistance, and the absence of a national legal framework were identified as the most pressing barriers. Given the existing workload pressures, most participants said that mentoring would be an added responsibility that cannot be sustained without formal policy support.

Theme 10: Stakeholder vision of an ideal mentorship model

A dual-pathway system, with tailored clinical mentorship structures for undergraduate nursing students and newly hired nurses, was the most commonly named preferred future system and suggested by most groups. Suggested duration ranged from 6 to 12 months, delivered through hybrid formats combining group and individual instruction. Recommended instructor-to-trainee ratios ranged from 1:2 to 1:5. National certification and periodic renewal were considered essential to ensure quality and accountability.

Theme 11: Student perspectives

Students emphasised the importance of consistent, trained clinical instructors throughout placements. As one student expressed: “We want mentors present, not just names on a schedule.”

Table 3 provides an overview of the themes and key findings from the national stakeholder focus group.

These descriptive patterns highlight the key strengths and challenges identified across the literature and the stakeholder consultation, providing a foundation for interpreting their implications in the Discussion.

## 4. Discussion

This work confirms that, despite Greece’s alignment with EU directives mandating theoretical and clinical training, a unified approach to clinical mentorship has not yet been established. Evidence from both the literature and the national stakeholder consultation highlights fragmented legislation, inconsistent terminology, and limited recognition of mentorship as a formal responsibility. These gaps contribute to variability in clinical learning experiences and place additional pressure on an already overstretched clinical workforce.

Findings from the literature and stakeholder input converge on the need for a dual-pathway approach that supports both undergraduate nursing students and newly hired nurses. International examples—including structured preceptorship systems in the United Kingdom, formal mentor certification in Finland, and university-embedded mentor preparation in The Netherlands—demonstrate that structured training, competency-based assessment, and both financial and non-financial incentives can strengthen mentorship capacity [4,9,23,30,31]. Slovenia’s national model further illustrates the value of coordinated governance: higher education institutions collaborate with clinical sites through shared responsibility across five dimensions, mentor qualification and training, student preparation, formal agreements, assessment and evaluation, and quality and safety [32]. These examples underscore that strong academic–clinical partnerships are central to maintaining consistent mentorship standards.

While these models offer transferable insights, stakeholders highlighted that adaptation must reflect Greece’s realities of chronic understaffing, high workload, and constrained institutional resources. For this reason, participants emphasised that mentorship structures must be embedded within existing professional development systems rather than added as additional duties without support. Stakeholders also agreed that consistent, structured processes—particularly standardised training, clear role expectations, and regular supervision—are urgently needed to reduce transition shock among newly qualified nurses and improve overall retention [15,23,24].

Across settings, non-financial incentives emerged as particularly relevant. Evidence shows that recognition systems, continuing-education credits, protected time for mentorship activities, and integrating mentorship into career progression pathways can substantially increase mentor engagement, especially where financial incentives are difficult to sustain [9,23]. Stakeholders similarly prioritised these strategies, noting that meaningful recognition and career relevance are often stronger motivators than small monetary rewards.

A persistent challenge identified through both evidence streams is the weak implementation of collaboration between universities and healthcare institutions. Although some agreements exist, accountability remains limited. Strengthening this interface will be essential for standardised mentor preparation, shared responsibility for student learning, and alignment between academic expectations and clinical realities.

Despite the value of these insights, several system-level constraints threaten sustainability. Limited hospital budgets restrict opportunities for dedicated mentorship time, while fragmented governance across education and health sectors impedes unified standards. Without national policy mechanisms to resource training, define mentorship roles, and coordinate academic–clinical collaboration, mentorship is likely to remain informal and inconsistently implemented.

### Policy Implications

The findings point to several feasible steps for strengthening clinical mentorship in Greece. First, national legislation should define mentor roles, certification requirements, and expectations for protected time, drawing on examples from Finland and the England, where such policies have enhanced accountability and workforce mobility [4,23,31]. Second, universities should embed mentorship pedagogy within existing curricula and collaborate with hospitals to develop joint training programmes. Evidence from The Netherlands and Slovenia suggests that university-led preparation improves instructional quality and standardisation [23,32]. Third, hospitals should incorporate mentorship into job descriptions, appraisal systems, and continuing professional development pathways. Non-financial incentives—such as recognition awards, CPD credits, structured supervision, or reduced night shifts—offer pragmatic, high-impact approaches in resource-limited environments. Finally, pilot programmes in selected hospitals could build local evidence, demonstrate feasibility, and strengthen political momentum for national adoption.

These considerations inform the conclusions and recommendations presented next.

## 5. Conclusions

This study mapped the strengths and gaps in clinical mentorship in Greece and compared them with selected European examples to identify stakeholder-driven priorities for reform. Drawing on findings from a narrative literature review and a national stakeholder focus group, the analysis revealed fragmented implementation, limited training and recognition for clinical instructors and mentors, and weak academic–clinical collaboration. These system shortcomings contribute to inconsistent clinical learning experiences and hinder the development of a coordinated national approach.

A dual-pathway mentorship model—addressing the distinct needs of undergraduate nursing students and newly hired nurses—emerged as a viable direction. Stakeholders highlighted the importance of structured preparation, national certification, protected time, and integrating mentorship responsibilities into career-progression frameworks. European experiences demonstrate that these approaches are feasible and can enhance educational quality, workforce retention, and professional development.

Strengthening clinical mentorship in Greece is both an educational priority and a patient safety imperative. Without coordinated national action, inconsistency in mentorship will continue to undermine workforce development and strain an already pressured health system. Three time-bound steps are recommended:

- Short-term (within 12 months): establish a national working group led by the Ministry of Health and academic partners to define standardised mentor roles, training requirements, and certification pathways.
- Medium-term (1–3 years): pilot structured mentor training and certification in selected regions, accompanied by monitoring of learning outcomes, workforce integration, and feasibility in current resource constraints.
- Long-term (3–5 years): integrate mentorship responsibilities and certification into national professional advancement frameworks to ensure sustainability and measurable improvements in educational quality, workforce stability, and patient safety.

Implementing these steps will help build a sustainable, nationally coordinated mentorship system capable of supporting Greece’s nursing workforce and improving patient care.

## Figures and Tables

**Table 1:** Key distinctions between mentorship, preceptorship, and clinical instruction.

**Term**	**Definition and** **Focus**	**Duration**	**Typical Context**	**Primary Goal**
Mentorship	Long-term, developmental relationship; includes career guidance, emotional support, professional growth	Months to years	Undergraduate programmes, career development	Broader identity formation, career support
Preceptorship	Time-limited, structured support to develop role-specific skills under direct supervision	Weeks to months	Transition into practice, new unit or specialty	Competence, confidence, safe transition
Clinical instruction	Integrative approach combining mentorship and preceptorship; often refers to supervision of students during placements	Ongoing during education	Undergraduate clinical placements	Skill acquisition, application of theory, safe patient care

**Table 2:** Summary of themes from the narrative literature review.

Theme	Focus	Key Findings
Theme 1. System-level factors in European nursing education and mentorship	Education reforms, harmonisation, workforce quality	EU reforms emphasise bachelor-level entry, structured mentorship models, and workforce mobility. Higher proportions of bachelor-prepared nurses are associated with reduced mortality, fewer adverse events, and better patient experiences
Theme 2. Definitions and conceptual variations in mentorship models	Use of terminology	Terms such as mentorship, preceptorship, and clinical instruction are inconsistently used. Mentorship is long-term and developmental; preceptorship is short-term and task-focused; clinical instruction often integrates elements of both. Inconsistent terminology complicates comparison across countries.
Theme 3: Transition to practice and early-career vulnerability	Support for new graduates	New graduates experience “transition shock.” Structured mentorship and transition programmes can improve resilience, reduce burnout, and increase retention.
Theme 4: Preparation and attributes of effective clinical instructors	Training and readiness	Effective clinical instructors require structured preparation, including communication skills, leadership, and adult-learning competence. Several European countries have formalised training, certification, and incentives for mentors.

**Table 3:** Summary of themes from the national stakeholder focus group.

Theme	Focus	Key Findings
Theme 5. Terminology and role clarity	Inconsistent definitions in practice	Terms such as mentor, clinical instructor, and coordinator are used inconsistently. Roles are informal and depend on individual initiative rather than structured assignment.
Theme 6. Support and training needs	Clinical instructor preparedness	Consultation findings on the need for a national training curriculum covering pedagogy, communication, leadership, simulation, and evaluation skills. Mentors feel underprepared without structured training.
Theme 7. Academic–clinical collaboration	Partnership gaps	Formal agreements exist between universities and hospitals but are inconsistently implemented. Stakeholders called for joint curricula, unified criteria for instructor selection, and shared accountability.
Theme 8. Incentives and recognition	Motivation and career development	Given resource constraints, non-financial incentives, such as recognition, continuing education credits, reduced night shifts, and career advancement, were prioritised over financial incentives.
Theme 9. Structural and organisational barriers	Systemic challenges	Understaffing, time constraints, cultural resistance, and lack of a national legal framework hinder consistent clinical mentorship delivery.
Theme 10. Vision for an ideal mentorship model	Preferred future system	Stakeholders favoured a dual-pathway mentorship model for students and newly hired nurses, with 6–12-month structures, instructor-to-trainee ratios of 1:2–1:5, hybrid delivery, national certification, and renewal requirements.
Theme 11. Student perspectives	Learning needs	Students emphasised the importance of consistent, trained clinical instructors.

## Data Availability

Data supporting the findings of this work are available from the corresponding author upon reasonable request. Due to the qualitative and policy-oriented nature of the focus group, full transcripts are not publicly available to protect participant confidentiality.

## Public Involvement Statement

No public involvement in any aspect of this research. Stakeholders contributed in their professional capacity.

## Guidelines and Standards Statement

This manuscript was developed in accordance with the Standards for Reporting Qualitative Research (SRQR) guidelines [33] which promote transparency and rigour in qualitative study design, data collection, analysis, and reporting. The SRQR framework was chosen as it aligns with the descriptive qualitative design and focus group methodology applied in this project.

## Abbreviations

The following abbreviations are used in this manuscript:

EU	European Union
RN	Registered Nurse
QCA	Qualitative Comparative Analysis
WHO	World Health Organization

## Appendix A

**Table A1:** List of 19 records included in the narrative review.

Ref. No.	Author, Year	Title	Summary of What the Publication Examines	Key Results, Main Findings
[3]	Mínguez Moreno I,; González de la Cuesta D.; Barrado Narvión M.J.; Arnaldos Esteban M.; González Cantalejo M.; 2023	Nurse mentoring: a scoping review	A scoping review mapping existing evidence on nurse-mentoring programmes, their structures, purposes, and reported outcomes across clinical settings.	Mentoring programmes consistently improved nurses’ competence, confidence, job satisfaction, and integration into practice, while reducing turnover and supporting organisational stability.
[4]	Irwin C.; Bliss J.; Poole K.; 2018	Does Preceptorship improve confidence and competence in Newly Qualified Nurses: A systematic literature review.	This paper is a systematic literature review that investigates whether preceptorship improves confidence and competence in newly qualified nurses. It analyzes published empirical studies to assess the effectiveness of preceptorship programmes during early career stages of nurses	The review concludes that one-to-one preceptorship can positively influence confidence and competence of newly qualified nurses, but due to limited empirical evidence overall, findings are not conclusive. The authors note that full preceptorship programmes (rather than informal preceptorship) seem to have greater impact.
[5]	Rush K.L.; Janke R.; Duchscher J.E.; Phillips R.; Kaur S.; 2019	Best practices of formal new graduate transition programmes: An integrative review.	The paper reviews empirical literature (2000–2018) on formal transition-to-practice programmes for newly graduated nurses, aiming to identify which programme components and structures consistently support successful transition to professional practice	Transition programmes that include formalised support (mentors/preceptors or designated resource persons), peer support, and structured orientation are associated with improved competence, critical thinking, confidence, and retention among new graduate nurses.“Bundled” strategies (i.e., combining mentoring, education, workplace support, and orientation) are more effective than isolated measures. Workplace environment and organisational commitment (staffing, support, realistic workload) influence how well new graduates adapt and integrate, good environment improves outcomes.
[7]	Keighley T.; 2009	The European Union standards for nursing and midwifery: information for accession countries revised and updated by Thomas Keighley, 2nd ed	Provides an overview of EU educational and professional standards for nursing and midwifery, including required competencies, training hours, and regulatory expectations.	EU nursing standards mandate harmonised education, defined clinical training requirements, and competency-based preparation to ensure workforce mobility and quality of care.
[8]	Collins S.; Hewer I.; 2014	The impact of the Bologna process on nursing higher education in Europe: A review.	The paper reviews how the Bologna Process has influenced nursing education reforms across Europe, focusing on degree structures, academic comparability, mobility, and the transition toward bachelor-level nursing education.	The Bologna Process led to greater alignment and transparency in nursing education systems, increased mobility opportunities for students and professionals, and strengthened the shift toward bachelor-level preparation. However, implementation varied widely between countries, and challenges remained in harmonising clinical training requirements and ensuring consistent quality across programmes.
[9]	Rafferty A.M.; Busse R.; Zander-Jentsch B.; Sermeus W.; Bruyneel L.; 2019	Strengthening health systems through nursing: Evidence from 14 European countries	Provides a comprehensive cross-country analysis of nursing workforce structures, education, regulation, roles, and working conditions across 14 European nations, examining how these factors influence health system performance.	Countries with well-educated nursing workforces, strong regulatory frameworks, and supportive work environments demonstrate better patient outcomes, higher quality of care, and improved workforce retention. The report emphasises the importance of investment in nursing education (including bachelor-level preparation) and clinical training quality to strengthen health systems.
[10]	Alspach J.G.; 2014	Nurse Education and Patient Mortality: Sorting Fact From Fury	The paper evaluates the evidence linking nurse education levels, particularly the proportion of bachelor-prepared nurses—to patient mortality outcomes. It analyses major studies, methodological issues, and the validity of commonly cited claims.	The author concludes that the evidence consistently shows an association between higher proportions of bachelor-educated nurses and reduced patient mortality, but emphasises the need for cautious interpretation due to methodological complexities. The paper supports strengthening educational standards while calling for improved study designs to clarify causality.
[11]	Aiken L.H.; Clarke S.P.; Cheung R.B.; Sloane D.M.; Silber J.H.; 2003	Educational Levels of Hospital Nurses and Surgical Patient Mortality	Investigates the relationship between the proportion of bachelor-prepared nurses in hospitals and surgical patient mortality rates.	Hospitals with higher percentages of bachelor-prepared nurses had significantly lower surgical mortality. A 10% increase in bachelor-educated nurses was associated with a 5% decrease in patient deaths. Demonstrates that nurse education level is a key factor in patient safety and outcomes.
[12]	Bruyneel L.; Li B.; Ausserhofer D.; Lesaffre E.; Dumitrescu I.; Smith H.L.; 2015	Organization of hospital nursing, Provision of Nursing Care, and Patient Experiences With Care in Europe	Analyzes how hospital nursing organisation, staffing, skill mix, workload, and work environment, affects the quality of nursing care and patient experiences across several European countries	Better-organised nursing environments, higher proportions of well-educated nurses, and adequate staffing were strongly associated with improved patient experiences, fewer reported care problems, and higher quality ratings. Demonstrates that organisational characteristics and nurse education significantly influence patient outcomes.
[14]	Benner P.; 1982	From Novice to expert	Introduces a developmental model describing how nurses progress from beginners to experts through experiential learning, clinical judgement development, and situational understanding.	Identifies five stages of skill acquisition (novice, advanced beginner, competent, proficient, expert). Demonstrates that expertise develops through experience, pattern recognition, and contextual understanding, not just formal education. The model highlights the need for strong clinical support and mentorship to accelerate competence development.
[15]	Duchscher J.E.B.; 2009	Transition shock: the initial stage of role adaptation for newly graduated Registered Nurses.	Explores the experiences of newly graduated nurses during their first months in practice, focusing on the emotional, cognitive, and professional challenges of transitioning from student to practicing nurse.	Identifies “transition shock,” a period marked by stress, uncertainty, role confusion, and steep learning demands. Highlights the need for structured support, mentorship, and realistic workplace expectations to ease this transition and reduce early-career attrition.
[21]	Aiken L.H.; Sloane D.M.; Bruyneel L.; Van Den Heede K.; Griffiths P.; Busse R.; 2014	Nurse staffing and education and hospital mortality in nine European countries: a retrospective observational study	Investigates how nurse staffing levels and nurse education (particularly the proportion of bachelor-educated nurses) affect hospital mortality across nine European countries.	Hospitals with better nurse staffing and higher proportions of bachelor-educated nurses had significantly lower mortality. A 10% increase in bachelor-prepared nurses was associated with a 7% reduction in 30-day inpatient mortality. Demonstrates that staffing and education are critical determinants of patient safety.
[22]	Race T.K.; Skees J.; 2010	Changing Tides: Improving Outcomes Through Mentorship on All Levels of Nursing	Explores how mentorship can enhance nursing performance and outcomes across different levels of nursing practice, from novice to experienced nurses.	Mentorship improves clinical skills, confidence, job satisfaction, and retention. The paper highlights that structured mentoring relationships contribute to better patient outcomes and strengthen professional development across the nursing workforce.
[23]	Dobrowolska B.; McGonagle I.; Kane R.; Jackson CS.; Kegl B.; Bergin M.; 2016	Patterns of clinical mentorship in undergraduate nurse education: A comparative case analysis of eleven EU and non-EU countries	Compares how undergraduate clinical mentorship is structured, implemented, and regulated across 11 European and non-European countries to identify differences, common patterns, and challenges.	Reveals substantial variation in mentorship models, including roles, preparation, workload, and assessment methods. Countries with formal national frameworks showed more consistency and better support for mentors and students. Highlights the need for clear standards, mentor training, and stronger academic–clinical collaboration.
[24]	Dahlke S.; Baumbusch J.; Affleck F.; Kwon J-Y.; 2012	The Clinical Instructor Role in Nursing Education: A Structured Literature Review	Reviews existing literature to define the role, responsibilities, and required competencies of clinical instructors in nursing education, and to identify factors that influence their effectiveness.	Effective clinical instructors possess strong communication skills, clinical expertise, teaching ability, and supportive interpersonal behaviours. Barriers identified include lack of formal preparation, competing workload demands, and limited institutional support. The review emphasises the need for structured training and recognition of clinical instructors.
[25]	Pasila K.; Elo S.; Kääriäinen M.; 2017	Newly graduated nurses’ orientation experiences: A systematic review of qualitative studies.	A systematic review synthesising qualitative research on how newly graduated nurses experience orientation programmes during their transition into professional practice.	New nurses’ orientation experiences varied widely. Positive experiences were linked to structured support, approachable preceptors, and clear guidance, whereas poor orientation led to stress, insecurity, and feeling unprepared. The review highlights the importance of well-organised, supportive orientation to promote smoother transition and retention.
[26]	Edwards D.; Hawker C.; Carrier J.; Rees C.; 2015	A systematic review of the effectiveness of strategies and interventions to improve the transition from student to newly qualified nurse	Systematically reviews interventions designed to support nursing students during their transition into the role of newly qualified nurses, evaluating which strategies are effective.	Structured support interventions—especially preceptorship, mentorship, extended orientation, and formal transition programmes improve confidence, competence, and adaptation. Evidence shows that programmes combining multiple strategies are more effective than single interventions. Highlights the importance of organisational support and protected time for learning.
[27]	Brook J.; Aitken L.; Webb R.; MacLaren J.; Salmon D.; 2019	Characteristics of successful interventions to reduce turnover and increase retention of early career nurses: A systematic review.	Systematically reviews interventions aimed at reducing turnover and improving retention among early-career nurses, analysing what makes these interventions effective.	Successful interventions commonly include structured transition programmes, mentorship, supportive work environments, and opportunities for professional development. Multi-component programmes were most effective. Strong leadership and organisational support were key determinants of successful retention.
[29]	Jahan F.; Sadaf S.; Kalia S.; Khan A.; Hamza HB.; 2008	Attributes of an effective clinical teacher: a survey on students’ and teachers’ perceptions.	Surveys nursing students and clinical teachers to identify which qualities and behaviours define an effective clinical teacher in nursing education.	Both students and teachers agreed that effective clinical teachers demonstrate strong clinical competence, good communication, supportive attitudes, professionalism, and the ability to provide constructive feedback. The study highlights the need to develop these attributes through training to improve clinical teaching quality.

## Appendix B

**Table A2:** Descriptive codebook example.

Meaningful Unit	Code Identification	Subthemes	Theme
“In my hospital, the mentor is whoever has time on the day; there is no consistency.” Stakeholder meeting 18 November 2024, Athens, Greece.	Lack of formal assignment process	Inconsistent mentor allocation; informal role definition	Terminology and role clarity
“Mentorship and preceptor definitions do not work in Greece, maybe we can call it supervision and instruction. There are no rules (for the name), everybody can do what they want.” Stakeholder meeting 18 November 2024, Athens, Greece.	Confusion in role terminology	Overlapping titles; absence of standard definitions	
“We cannot continue without structured training; mentors need the tools to guide students properly” Stakeholder meeting 18 November 2024, Athens, Greece	Need for structured preparation	Mentor readiness; pedagogical training requirements	Support and training needs
“Some professionals have a negative attitude towards teaching and mentoring, due to fear of losing time for clinical work”. Stakeholder meeting 18	Competing workload pressure	Time constraints; low motivation for mentorship	

## Appendix C

**Table A3:** European countries included in the narrative review.

Country/Region	Regulatory or Structural Framework	Clinical Mentor Preparation	Supervision Model
EU/European Region (general) [7,8,9,16]	EU Directive 2013/55/EU and Bologna Process shape minimum education standards and competency expectations across Europe.	Preparation requirements vary, but many countries adopt competency-based frameworks aligned with EU directives.	Structured clinical placements with defined learning outcomes; assessment responsibilities regulated nationally or institutionally.
Greece [17,18,19,20]	No unified national framework; fragmented regulation across institutions and ministries.	No national requirement for pedagogical training; mentor preparation varies significantly.	Supervision structures inconsistent; clinical instructor roles often informal or undefined.
England (United Kingdom) [26,27,30,31]	National Preceptorship Framework and NMC supervision and assessment standards guide clinical mentorship.	Mandatory preparation for supervisors and assessors; formal curricula and organisational endorsement required.	Distinct practice supervisor and practice assessor roles; structured supervision ratios and assessment processes.
Finland [25,34]	National laws regulate nursing education; clinical placements embedded in higher education quality systems.	Pedagogical training frequently required (often at Master’s level); national expectations for instructor competencies.	Strong academic–clinical integration with joint assessment responsibilities.
The Netherlands [9,35]	National accreditation frameworks define standards for clinical education and mentorship across institutions.	Instructor training provided by employers; structured programmes include pedagogy, coaching, and assessment.	Standardised supervision models across clinical sites; mentorship integrated into national quality frameworks.
Denmark [9,35]	National regulations define the clinical instructor role, qualifications, and required training.	Certified instructor courses typically mandatory; often delivered through universities or regional centres.	Clear supervision ratios and strong academic–clinical coordination.
Slovenia [32,34]	National guidelines detail competencies and clinical training expectations for undergraduate nursing programmes; evidence-based recommendations guide new-graduate induction.	Strong emphasis on structured mentor preparation, including pedagogical and supervisory competencies.	Formal induction pathways for newly graduated nurses; clearly defined mentorship timelines and expectations.
